# Supplementary material for: The Detection of Immunity against WT1 and SMAD4P130L of EpCAM+ Cancer Cells in Malignant Pleural Effusion
Source: Int J Mol Sci. 2022 Oct 12;23(20):12177. doi: 10.3390/ijms232012177 (PMC9602695; doi:10.3390/ijms232012177)
Supplement: Supplementary file 1 [file ijms-23-12177-s001.zip › ijms-1913863-supplementary.pdf]

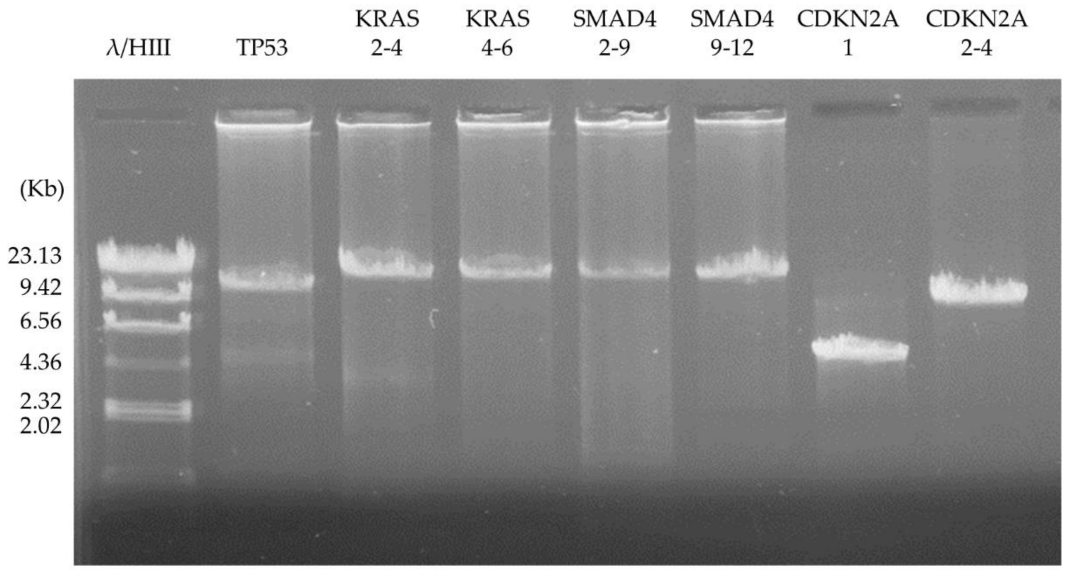

Supplementary Figure S1. Pancreatic cancer Big 4 Genes Long PCR.
The agarose gel electrophoresis results of long-range PCR products targeting KRAS, CDKN2A, TP53, and SMAD4 using primers (Supplemental Table1). The PCR products were subjected to each indicated lane.

Materials and Methods

Identification of the *SMAD4* P130L mutation

Genomic DNA was extracted from two MPE samples and peripheral blood mononuclear Cells (PBMCs) using a rapid extraction method [35]. The long-range PCR primers used in this study were designed by Primer3 v.0.4.0 (http://bioinfo.ut.ee/primer3–0.4.0/ last accessed July 27, 2022) [36], using the following parameters: primer length, 26–27–30 mer; Tm, 67°C–67.5°C–68°C; Max Tm difference, 0.1°C; and GC%, 45–50–60. GC Clump 2 and other parameters were used with the default setting. Each PCR reaction contained 1 μl of 20 ng/μl genomic DNA in a 10 μl reaction volume and each long PCR primer sequence; the final concentrations are shown in Supplemental Table 3. Touch–down PCR cycles were performed with KOD One DNA polymerases (TOYOBO, Co., Ltd., Osaka, Japan) under the following conditions: 5 cycles of 98°C for 10 s and 74°C for 10 min; 5 cycles of 98°C for 10 s and 72°C for 10 min; 5 cycles of 98°C for 10 s and 70°C for 10 min; and 25 cycles of 98°C for 10 s and 68°C for 10 min.

Long PCR products were purified using a High Pure PCR Product Purification Kit (Roche Diagnostics GmbH, Mannheim, Germany). According to the manufacturer's protocol, an NGS library was prepared using a Nextera XT DNA Library Prep kit (Illumina, San Diego, CA, USA). The libraries were quantified using an HS Qubit dsDNA assay (Thermo Fisher Scientific, Waltham, MA, USA) and a TapeStation 4200. Qualified size distributions were checked on a TapeStation 4200 using High Sensitivity D1000 ScreenTape. A 12.5 pM library was sequenced on an Illumina MiSeq system (2 × 150 cycles) according to the standard Illumina protocol (Illumina).

The FASTQ files were generated using the bcl2fastq software (Illumina). The FASTQ files were aligned to the reference human genome (hg38) using the Burrows Wheeler Aligner MEM algorithm (BWA–MEM version 0.7.17–r1188) [37]. Mosaic mutations were identified using GATK's Mutect2 (Version 4.0.6.0) [38,39]. The SNVs and INDELs were functionally annotated by SnpEff (Version 4.3t) to classify each variant into a functional class (HIGH, MODERATE, LOW, and MODIFIER) [40]. The Database of Short Genetic Variations dbSNP (Version 151) and ClinVar were used for variant annotation [41,42].

References

35. Lahiri DK, Schnabel B. DNA isolation by a rapid method from human blood samples: effects of MgCl2, EDTA, storage time, and temperature on DNA yield and quality. Biochem Genet. 1993;31(7–8):321–328. doi:10.1007/BF02401826

36. Untergasser A, Cutcutache I, Koressaar T, Ye J, Faircloth BC, Remm M, Rozen SG. Primer3--new capabilities and interfaces. Nucleic Acids Res. 2012;40(15). doi:10.1093/NAR/GKS596
37. Li H, Durbin R. Fast and accurate long-read alignment with Burrows-Wheeler transform. Bioinformatics. 2010;26(5):589–595. doi:10.1093/BIOINFORMATICS/BTP698
38. Cibulskis K, Lawrence MS, Carter SL, Sivachenko A, Jaffe D, Sougnez C, Gabriel S, Meyerson M, Lander ES, Getz G. Sensitive detection of somatic point mutations in impure and heterogeneous cancer samples. Nat Biotechnol. 2013;31(3):213–219. doi:10.1038/NBT.2514
39. Wilm A, Aw PPK, Bertrand D, Yeo GHT, Ong SH, Wong CH, Khor CC, Petric R, Hibberd ML, Nagarajan N. LoFreq: a sequence-quality aware, ultra-sensitive variant caller for uncovering cell-population heterogeneity from high-throughput sequencing datasets. Nucleic Acids Res. 2012;40(22):11189–11201. doi:10.1093/NAR/GKS918
40. Cingolani P, Platts A, Wang LL, Coon M, Nguyen T, Wang L, Land SJ, Lu X, Ruden DM. A program for annotating and predicting the effects of single nucleotide polymorphisms, SnpEff: SNPs in the genome of Drosophila melanogaster strain w1118; iso-2; iso-3. Fly (Austin). 2012;6(2):80–92. doi:10.4161/FLY.19695
41. Sherry ST, Ward MH, Kholodov M, Baker J, Phan L, Smigielski EM, Sirotkin K. dbSNP: the NCBI database of genetic variation. Nucleic Acids Res. 2001;29(1):308–311. doi:10.1093/NAR/29.1.308
42. Landrum MJ, Lee JM, Riley GR, Jang W, Rubinstein WS, Church DM, Maglott DR. ClinVar: public archive of relationships among sequence variation and human phenotype. Nucleic Acids Res. 2014;42(Database issue). doi:10.1093/NAR/GKT1113

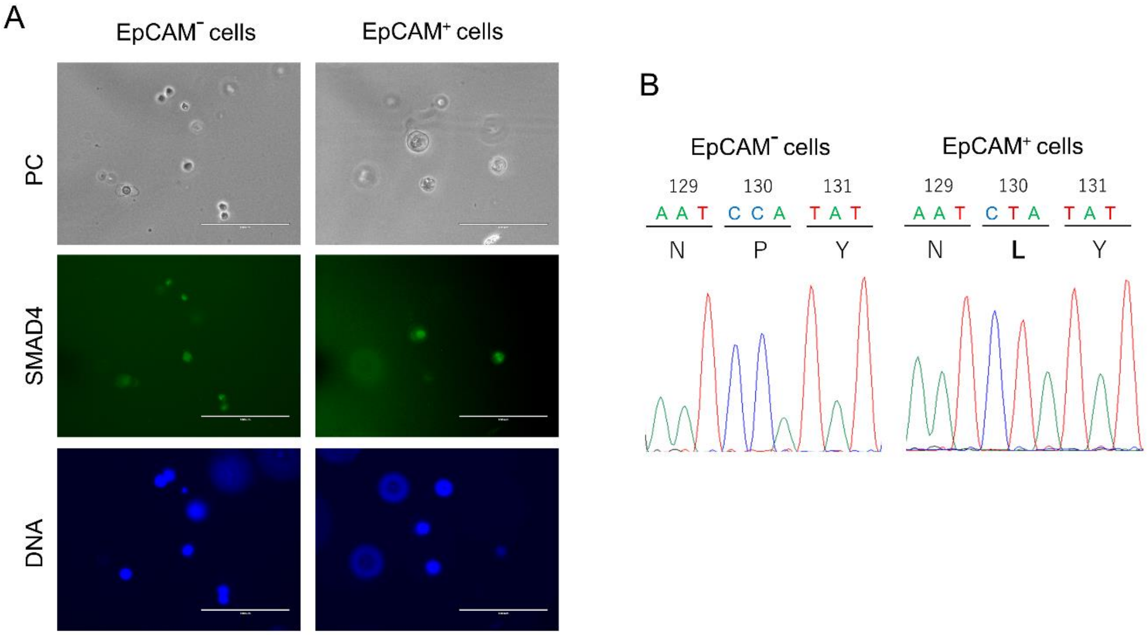

Supplementary Figure S2. A homogenous SMAD4^{P130L} expression on EpCAM^{+} cancer cells in MPE. (A) Expression of SMAD4 on EpCAM positive or negative cells sorted from whole cells in MPE^{1st}. PC: phase contrast; SMAD4: cells were stained with anti–SMAD4 antibody conjugated with Alexa Fluor® 488; DNA: DAPI staining. The white bar indicates 100 μm. (B) Direct PCR sequencing for SMAD4 Pro130Leu missense mutation. Bolded amino acid one letter code revealed missense mutation.

Materials and Methods

Immunofluorescence staining

EpCAM positive and negative live cells, sorted from MPE samples using a Cell Sorter SH800 (Sony Biotechnology Inc., Tokyo, Japan), were examined for WT1 or SMAD4 expression. Immediately after sorting, a cell slide was prepared using a Smear Gell kit (GenoStaff Co., Ltd., Tokyo, Japan) according to the manufacturer's instructions to observe those expressions in an environment approximating MPE. Subsequently, 1 × 10^5 cells were fixed with a 10% formalin neutral buffer solution (Wako Pure Chemicals Ltd., Osaka, Japan) for 30 min and then rinsed three times with PBS. The cells were then permeabilized with a 0.1% Triton X–100 (Sigma–Aldrich Co. LLC, St. Louis, MO, USA) solution in PBS at room temperature for 5 min and blocked with UltraCruz Blocking Reagent (Santa Cruz Biotechnology, Inc., Dallas, TX, USA) for 60 min at room temperature. After blocking, cells were incubated with anti–SMAD4 rabbit monoclonal antibody–C–terminal Alexa Fluor® 488 (1:100, clone SP306, Abcam, Inc., Cambridge, UK) at 4℃ for 16 hr. Finally, the cells were rinsed with PBS and incubated with DAPI (Thermo Fisher Scientific) at 300 nM in PBS at room temperature for 2 min to stain the nuclei. The slides were imaged using fluorescence microscopy (EVOS® FL Cell Imaging System; Thermo Fisher Scientific).

Direct PCR sequencing

In brief, total RNA was isolated using the RNeasy Mini Kit (Qiagen GmbH, Hilden, Germany) and generated complementary DNA (cDNA) using SuperScript III Reverse Transcriptase (Thermo Fisher Scientific). PCR reaction performed using the designed primers for SMAD4 amplification (Forward: 5′– CAAATGGAGCTCATCCTAG –3′; Reverse: 5′– TGTATGTCTCTGTCGATGC–3′; purchased from Eurofins Genomics K.K., Tokyo, Japan) as follows: 2 min initial denaturation at 94 °C, followed by 30 cycles of 10-sec denaturation at 98 °C, 30-sec primer annealing at 57 °C and 30-sec extension at 68 °C, followed by a final extension period of 7 min at 68 °C. The PCR product was purified using PCR purification Kit (Qiagen), and then sequencing was achieved using Big Dye Terminator v3.1 and an ABI automatic sequence analyzer (model 3500xL; Thermo Fisher Scientific) with primers for SMAD4 sequencing (Forward: 5′–TGGTCGGAAAGGATTTCC –3′; Reverse: 5′– TTCAGTGGACAACGATGG –3′; Eurofins Genomics).

Supplementary Table S1. Long PCR Primers for TP53, KRAS, SMAD4, and CDKN2A

Primer Name	Primer Seq			Primer final conc. (uM)	Chr.	GRCh38 position		Product size (bp)
TP53_L–2–11FN	5'–	ccagcactttcctcaactctacatttcc	–3'	0.075	17	7679663	7679636	12621
TP53_L–2–11R	5'–	gagatctgcagagtaggtccaagtgtcc	–3'		17	7667042	7667069	
KRAS_L–2–4FN	5'–	acacttagaggtgggggtccactagg	–3'	0.075	12	25245857	25245832	23161
KRAS_L–2–4RN	5'–	acccaaggccacaaaactgtataaaacc	–3'		12	25222697	25222724	
KRAS_L–4–6FN	5'–	ggtcaagaggagtacagtgcaatgagg	–3'	0.075	12	25227346	25227320	23960
KRAS_L–4–6R	5'–	aacctgcttaactcacccagtttctgc	–3'		12	25203386	25203412	
SMAD4_L–2–9FN	5'–	cctcccttacttcaggctctgttaccc	–3'	0.15	18	51042388	51042414	24126
SMAD4_L–2–9R	5'–	ggtaatttgccaaagtcacatgacagc	–3'		18	51066513	51066487	
SMAD4_L–9–12F	5'–	tctgaagtgttcttcccaatcatctgc	–3'	0.075	18	51064329	51064355	21348
SMAD4_L–9–12RN	5'–	cagaaggcagagagacacacaactgc	–3'		18	51085677	51085652	
CDKN2A_L–1F	5'–	taggtgcagaggaagacccataaaagg	–3'	0.15	9	21996649	21996623	4857
CDKN2A_L–1R	5'–	ttctgaaagggctatggttcacttgg	–3'		9	21991793	21991818	
CDKN2A_L–2–4F	5'–	ttctcccccgtccgtattaaataaacc	–3'	0.15	9	21975691	21975665	9196
CDKN2A_L–2–4R	5'–	ggctctatgtccagaggaactcatcg	–3'		9	21966496	21966521	

Supplementary Table S2. Antibodies for analysis of MPE samples

Reagent	Clone	Source
Alexa Fluor 488 or APC-conjugated anti-CD326 (EpCAM)	9C4	BioLegend
FITC-conjugated anti-CD274 (PD-L1)	MIH1	BD Biosciences
PE or APC-Cy7-conjugated anti-CD366 (TIM-3)	F38-2E2	BioLegend
BV510-conjugated anti-CD45	2D1	BioLegend
FITC-conjugated anti-CD3	SK7	BD Biosciences
APC-Cy7-conjugated anti-CD8	SK1	BioLegend
Pacific Blue-conjugated anti-CD279 (PD-1)	EH12.2H7	BioLegend
APC-conjugated anti-CD14	TÜK4	Miltenyi Biotec
PE-conjugated anti-CD68	Y1/82A	Thermo Fisher Scientific
BV421-conjugated anti-CD163	GHI/61	BD Biosciences

Supplementary Table S3. Reagents for memory T cell subsets of WT1-CTLs

Reagent	Clone	Source
FITC-conjugated anti-CD3	SK7	BD Biosciences
APC-Cy7-conjugated anti-CD8	SK1	BioLegend
WT1 Tetramer-CYTWNQMNL-PE	N/A	Medical and Biological Laboratories
PerCP-Cy5.5-conjugated anti-CD62L	DREG-56	BioLegend
BV421-conjugated anti-CD45-RO	UCHL1	BioLegend
